# Psychological Therapy Quantity and Depressive Symptom Reduction in Psychedelic-Assisted Therapy: A Systematic Review and Meta-Analysis

**DOI:** 10.1001/jamanetworkopen.2025.54843

**Published:** 2026-01-21

**Authors:** Gianluca Andri Florineth, Isabell Klima, Anna Laura Boeker, Polina Catzeflis, Alexander Wopfner, Niklaus Denier, Tobias Bracht, Kristina Adorjan, Mario Pfammatter, Felix Müller, Leila Maria Soravia

**Affiliations:** 1 Translational Research Center, University Hospital of Psychiatry and Psychotherapy, University of Bern, Bern, Switzerland; 2 Graduate School for Health Sciences, University of Bern, Bern, Switzerland; 3 Department of Psychiatry (UPK), University of Basel, Basel, Switzerland; 4 Südhang Clinic, Kirchlindach, Switzerland; 5 University Hospital Basel, Division of Clinical Pharmacology and Toxicology, Department of Biomedicine and Department of Clinical Research, University of Basel, Basel, Switzerland

## Abstract

**Importance:**

Psychedelic-assisted therapy (PAT) is a novel intervention for depressive symptoms, typically delivered with additional psychological therapy sessions. Quantitative evidence on how this concomitant therapy contributes to treatment outcomes is limited, highlighting the need for a systematic synthesis.

**Objective:**

To evaluate whether the quantity of psychological therapy is associated with symptom reduction in PAT for depressive symptoms.

**Data Sources:**

PubMed, PsycINFO, and Scopus databases were searched from inception to June 16, 2025.

**Study Selection:**

The analysis included controlled clinical trials involving adults with depressive symptoms who received PAT using classic serotonergic psychedelics (eg, psilocybin or lysergic acid diethylamide). Within these trials, psychedelic dosing sessions were embedded in therapeutic sessions before (preparation) and after (integration). Studies were excluded if they used microdosing as the primary intervention, involved naturalistic or purely pharmacological administration, or did not report therapy session count or duration. Qualitative studies, reviews, case reports, and conference abstracts were also excluded. Of the 226 records identified, 42 full texts were assessed by 2 independent reviewers, of which 12 met inclusion criteria.

**Data Extraction and Synthesis:**

Two reviewers independently extracted data and assessed risk of bias according to Preferred Reporting Items for Systematic Reviews and Meta-Analyses guidelines. Multilevel random-effects meta-analysis and multilevel metaregressions were conducted using robust variance estimation.

**Main Outcomes and Measures:**

The primary outcomes were standardized mean differences (Hedges *g*) in depressive symptoms at all available posttreatment time points. Metaregression analyses assessed associations of different metrics of psychological therapy quantity (duration in hours, number of sessions, and total duration in weeks) with treatment outcomes.

**Results:**

The 12 included trials had a total sample of 733 participants (365 female [49.8%]; mean [SD] age, 43.1 [6.2] years). PAT showed a large overall effect size in reducing depressive symptoms compared with control conditions (Hedges *g* = −0.84; 95% CI, −1.15 to −0.54; *P* < .001). More preparation therapy hours were significantly associated with greater symptom reduction (β = −0.13; 95% CI, −0.24 to −0.01; *P* = .04). No significant associations were found for hours of postdosing integration (β = −0.02; 95% CI, −0.08 to 0.05; *P* = .53) or total session count (β = −0.01; 95% CI, −0.09 to −0.08; *P* = .86). Longer follow-up periods, measured in weeks from substance administration, were generally associated with smaller treatment effect sizes (β = 0.02; 95% CI, 0.01 to 0.04; *P* = .003). Risk of bias was high in the majority (9 [75%]) of studies, mostly due to ineffective blinding.

**Conclusions and Relevance:**

In this systematic review and meta-analysis of controlled clinical trials investigating PAT for depressive symptoms, a greater quantity of preparation therapy was associated with significantly larger reductions of depressive symptoms. These findings highlight a potential key role of preparation in optimizing PAT outcomes. The observed association primarily reflects quantitative aspects of therapy exposure rather than qualitative or process-related dimensions of the therapeutic interaction. Additional systematic research is needed to clarify this association and optimize all therapeutic components within psychedelic interventions.

## Key Points

**Question:**

Is the quantity of psychological therapy associated with symptom reduction in psychedelic-assisted therapy (PAT) for depressive symptoms?

**Findings:**

In this systematic review and meta-analysis of 12 controlled clinical trials with 733 participants, a greater amount of preparatory psychological therapy was associated with a larger reduction of depressive symptoms following psychedelic administration. Total duration of psychological therapy as well as quantity of postpsychedelic integration sessions were not significantly associated with symptom reduction.

**Meaning:**

These findings suggest that preparation sessions before psychedelic administration may play an important role in optimizing treatment outcomes in PAT.

## Teaser

This systematic review and meta-analysis examines the association between quantity of psychological therapy and symptom reduction in psychedelic-assisted therapy for depressive symptoms.

## Introduction

Depression affects approximately 300 million people worldwide and places an immense burden on individuals, communities, and health care systems.^1^ Despite effective pharmacological and psychological treatments, approximately one-third of patients with depression remain resistant to treatment, highlighting a need for new treatment approaches.^2^

Psychedelic-assisted therapy (PAT) has seen a resurgence of scientific and public interest as a potential treatment option for depressive symptoms, with clinical trials showing large and rapid reductions in symptoms after just 1 or 2 dosing sessions embedded in psychological therapy.^3,4,5,6^ Currently, PAT treatment paradigms almost always follow 3 distinct phases: preparation, dosing, and integration sessions.^7,8^ Although this combination of pharmacological and psychological therapy is almost universally employed in PAT protocols, most research to date has been primarily focused on the safety and efficacy of the psychedelic substance itself, leaving the psychological therapy components poorly understood and without any clear guidelines.^9,10^

This lack of systematic research has led to uncertainty and heated debate. Some researchers and stakeholders questioned the necessity of psychological therapy in general and argue that the psychedelic experience alone is sufficient to drive therapeutically meaningful change.^11,12^ These uncertainties are mirrored in recent regulatory decisions, as seen in the US Food and Drug Administration’s rejection of 3,4-methylenedioxymethamphetamine–assisted therapy. This rejection was partly due to uncertainties about psychological therapy’s role in treatment efficacy and a lack of standardized treatment protocols overall.^13,14^

As PAT moves toward broader clinical adoption and increasing regulatory scrutiny, it will be essential to clarify the role of psychological therapy in PAT. This systematic review and meta-analysis synthesizes all current controlled trials on PAT for depressive symptoms, focusing on how psychological therapy amount—measured by session number and duration—affects treatment outcomes. Our main goal is to offer novel insights into the role of psychological components in PAT and serve as a hypothesis-generating base toward future research.

## Methods

### Literature Search and Eligibility Criteria

This meta-analysis was conducted in accordance with the Preferred Reporting Items for Systematic Reviews and Meta-Analyses (PRISMA) reporting guideline.^15^ A comprehensive search of the databases PubMed, PsycINFO, and Scopus was conducted from database inception through June 16, 2025 to identify studies examining the outcomes of PAT for depressive symptoms. Reference lists of eligible articles and relevant reviews were also screened. Two reviewers (G.A.F. and I.K.) independently screened all titles and abstracts, followed by full-text review of potentially eligible studies. Disagreements were resolved through discussion within the research group. Searches were limited to peer-reviewed journal articles published in English or German. Gray literature, study protocols, and reports without results were excluded.

Studies were eligible for inclusion if they investigated adults with depressive symptoms and used classic serotonergic psychedelics (eg, psilocybin, lysergic acid diethylamide [LSD], or N, N-dimethyltryptamine [DMT]) for treatment. Studies had to use a sufficiently high dose to reliably elicit psychedelic experiences (defined as psilocybin >10 mg, LSD >50 μg, or DMT >15 mg intravenous bolus).^16^ Interventions had to occur within a formal therapeutic framework, encompassing psychological therapy sessions prior to dosing and integration sessions following dosing. The term *psychological therapy* also included studies where authors used the term *psychological support*.

Only controlled clinical trials were eligible. Studies were excluded if they used microdosing as the primary intervention, involved naturalistic or purely pharmacological administration, or did not report therapy session count or duration. Qualitative studies, reviews, case reports, and conference abstracts were also excluded. The review was not preregistered in a public registry. All inclusion criteria, outcomes, and analytic approaches were predefined and applied consistently (eMethods in Supplement 1).

### Data Extraction

Data were extracted independently by 2 reviewers (G.A.F. and I.K.) using a coding sheet in tabular form. Extracted data included depression scores at baseline and all reported follow-up time points after the last dosing session (mean or mean change and SD), the number and duration (in hours) of psychological therapy sessions, number of dosing sessions, total treatment duration (in weeks), sample demographics, diagnostic criteria.

Dosing sessions were distinguished from psychological therapy sessions for methodological reasons. Although dosing sessions constitute a core component of the psychotherapeutic process, their duration is largely determined by the pharmacokinetics of the psychedelic substance and show limited variability across trials. In contrast, preparatory and integration therapy hours vary substantially between studies. These adjunctive therapeutic components were, therefore, the focus of our analyses, given their potential importance and the observation that recent discussions of PAT have placed disproportionate emphasis on the pharmacological dosing sessions.^11,17^ If a study compared more than 1 treatment condition with a single control group, the control group was split to avoid multiplicity^18^ (eMethods in Supplement 1).

### Risk of Bias

Risk of bias was assessed independently by 2 reviewers (G.A.F. and I.K.). For randomized trials, the Cochrane Risk of Bias 2 tool^19^ was used. For nonrandomized studies, we used the Risk of Bias in Nonrandomized Studies of Interventions tool.^20^ Discrepancies in bias assessment were resolved through discussion within the research group.

### Publication Bias

Potential publication bias was evaluated through visual inspection of funnel plot asymmetry and formally tested using the Egger regression test. To estimate the potential impact of missing studies on the overall effect size, the trim-and-fill method was used.^21^ The adjusted pooled estimate was computed after imputing missing studies to assess the robustness of the primary findings.

### Statistical Analysis

To synthesize effect sizes across studies and account for dependent effect sizes within studies, a multilevel meta-analysis was conducted using the metafor package^22^ (version 4.4) in R version 4.4.2 (R Project for Statistical Computing). Two-sided *P* < .05 was considered significant. The primary outcome was the standardized mean difference (Hedges *g*) in depressive symptom severity between treatment and control conditions, calculated at each reported follow-up time point. A random-effects model with a hierarchical structure was specified: effect sizes were nested within studies, allowing for shared variance within clusters due to repeated measurements. To address statistical dependence among effect sizes derived from repeated measures, an approximated variance-covariance matrix combined with robust variance estimation (RVE) was applied to all models.^23^ RVE provides valid SEs and test statistics under conditions of unknown or misspecified within-study covariance structures, thereby enhancing the reliability of inference.^24^ All models were estimated using restricted maximum likelihood.

To examine whether study-level psychological therapy quantity was associated with the magnitude of treatment effect sizes, a series of univariable metaregression models were conducted. Psychological therapy was quantified in multiple ways: amount of preparation and integration sessions (in hours), total amount of psychological therapy (in hours), number of nondosing therapy sessions, and total duration (in weeks). Additional exploratory study-level covariates included mean participant age, sex, study year, duration of follow-up (in weeks), number of dosing sessions, clinician-rated vs self-rated depression scales, and baseline symptom severity (categorical). Each model included a single covariate, while retaining the full random-effects and dependency structure. Due to the limited number of contributing studies, multivariate metaregression was not conducted to avoid overfitting and unstable parameter estimation.^25^

Heterogeneity between and within studies was quantified using the *I*^2^ measure. Multiple sensitivity analyses were performed to assess the robustness of our results. To assess the influence of within-study dependence assumptions (φ), all models were re-estimated using a range of values. We computed Cook distances to identify influential studies, and refit models after removing influential outliers. As the included trials were heterogeneous in their control conditions, we also conducted a sensitivity analysis excluding studies with waiting list and active treatment control condition, as they may lead to an overestimation or underestimation of treatment outcomes.

## Results

### Study Selection and Characteristics

**Figure 1.**
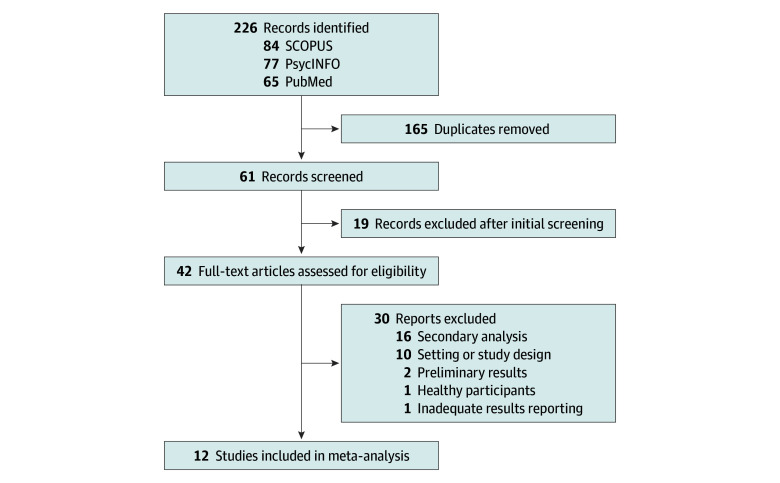
PRISMA Flowchart of Literature Search

The systematic search yielded 226 records, of which 214 were excluded (Figure 1). In total, 12 studies met our inclusion criteria,^3,4,5,6,26,27,28,29,30,31,32,33^ contributing a total of 36 effect sizes from different follow-up time points (total sample of 733 participants; 365 female [49.8%]; mean [SD] age, 43.1 [6.2] years). Across these studies, PAT was administered using psilocybin (10 studies)^3,4,5,26,27,28,29,30,32,33^ and LSD (2 studies).^31,6^ All used PAT protocols including psychological therapy sessions before and after substance administration. Trials assessed patients with major depressive disorders (8 studies),^3,4,5,6,28,29,30,32^ depressive symptoms related to life-threatening illness and anxiety (3 studies),^26,27,31^ and moderate-to-severe depressive symptoms related to frontline pandemic work (1 study).^33^

Total preparation ranged from 1 to 8 hours (mean [SD], 3.7 [2.3]), total integration from 2.5 to 12 hours (mean [SD], 4.4 [2.2]), and total number of nondosing sessions from 3 to 9 (mean [SD], 5.8 [1.9]). Follow-up assessments spanned 1 to 16 weeks (mean [SD], 4.8 [3.9]). Sample characteristics are summarized in the Table.

**Table.** Characteristics of All Included Trials Using Psychedelics for the Treatment of Depressive Symptoms

Study	Design	No. of participants	Symptom description	Age, mean (SD), y	Female, No. (%)	Intervention		Outcome assessment^a^	Preparation time, mean, h^b^^,^^c^	Integration time, mean, h^b^^,^^c^	Therapy sessions, No.^c^
						Treatment	Control				
Griffiths et al,^26^ 2016	Randomized, double-blind, crossover trial	51	Depressive symptoms due to life-threatening illness	56.3 (10)	25 (49)	Psilocybin, 22 or 30 mg/70 kg; 1 dosing session	Psilocybin, 1 or 3 mg/70 kg; 1 dosing session	HAM-D, 5 wk after	8	4.5	7
Ross et al,^27^ 2016	Double-blind, placebo-controlled, crossover trial	29	Depressive symptoms due to life-threatening illness	56.3 (12.9)	18 (62)	Psilocybin, 0.3 mg/kg; 1 dosing session	Niacin, 250 mg; 1 dosing session	BDI, 2, 6, and 7 wk after	6	2.5	9
Carhart-Harris et al,^3^ 2021	Double-blind RCT	59	Major depressive disorder	41.2 (10.7)	20 (33.9)	Psilocybin, 25 mg; 2 dosing sessions	Psilocybin 1 mg plus escitalopram 10 mg; 2 dosing sessions	QIDS, 1, 2, and 3 wk after	3	Not reported	Up to 9
Davis et al,^28^ 2021	Double-blind, waiting list; RCT	27	Major depressive disorder	39.8 (12.2)	11 (40.7)	Psilocybin, 20 + 30 mg/70 kg; 2 dosing sessions	Waiting list	HAM-D, 1 and 4 week after	8	2.5	4
Goodwin et al,^4^ 2022	Double-blind RCT	233	Treatment-resistant depression	39.8 (12.2)	121 (51.9)	Psilocybin, 10 mg or 25 mg; 1 dosing session	Psilocybin, 1 mg; 1 dosing session	MADRS, 1, 3, 6, 9, and 12 wk after	2	2.5	Approximately 5
von Rotz et al,^29^ 2022	Double-blind RCT	52	Major depressive disorder	36.8 (10.4)	33 (63.4)	Psilocybin, 15 mg; 1 dosing session	Mannitol, 1 dosing session	MADRS, 1 and 2 wk after	2	3	5
Raison et al,^5^ 2023	Double-blind RCT	104	Major depressive disorder	41.1 (11.3)	52 (50)	Psilocybin, 25 mg; 1 dosing session	Niacin, 100 mg; 1 dosing session	MADRS, 1, 2, 4, and 6 wk after	7	4	Approximately 4
Sloshower et al,^30^ 2023	Placebo-controlled, double-blind, fixed-order study	19	Major depressive disorder	42.8 (13.8)	13 (69.4)	Psilocybin, 0.3 mg/kg; 1 dosing session	Cellulose, 1 dosing session	HAM-D, 1 and 2 wk after	4	4	6
Holze et al,^31^ 2023	Randomized, double-blind, placebo-controlled, crossover trial	42	Depressive symptoms in patients with anxiety with or without life-threatening disease	45 (12)	20 (47.6)	LSD, 200 μg; 2 dosing sessions	LSD, 20 μg; 2 dosing sessions	HAM-D, 2, 8, and 16 wk after	1	4	5
Back et al,^33^ 2024	Double-blind RCT	30	Depressive symptoms related to frontline pandemic work	38 (NA)	15 (50)	Psilocybin, 25 mg; 1 dosing session	Niacin, 100 mg; 1 dosing session	MADRS, 4 wk after	2.5	3.75	5
Rosenblat et al,^32^ 2024	Randomized, waiting list-clinical trial	31	Treatment-resistant depression	44.4 (13.7)	12 (38.7)	Psilocybin, 25 mg; 1 dosing session	Waiting list	MADRS, 1 and 2 wk after	1.5	3	3
Müller et al,^6^ 2025	Double-blind RCT	56	Major depressive disorder	40.7 (12.6)	25 (44.6)	LSD, 100 + 200 μg; 2 dosing sessions	LSD, 25 + 25 μg; 2 dosing sessions	IDS-C and IDS-SR, 2, 6, and 12 wk after	4	8	6

Abbreviations: BDI, Beck Depression Inventory; HAM-D, Hamilton Depression Rating Scale; IDS-C, Inventory of Depressive Symptomatology Clinician-Rated; IDS-SR, Inventory of Depressive Symptomatology Self-Report; LSD, lysergic acid diethylamide; MADRS, Montgomery-Åsberg Depression Rating Scale; NA, not applicable; QIDS, Quick Inventory of Depressive Symptomatology; RCT, randomized clinical trial.

^a^ Outcome assessment refers to the time in weeks after the last substance administration.

^b^ If session duration was provided as a range, the mean is shown (eg, 1 to 1.5 h = 1.25 h).

^c^ Reported session count and duration are at the primary study end point.

### Overall Association of PAT With Depressive Symptoms

**Figure 2.**
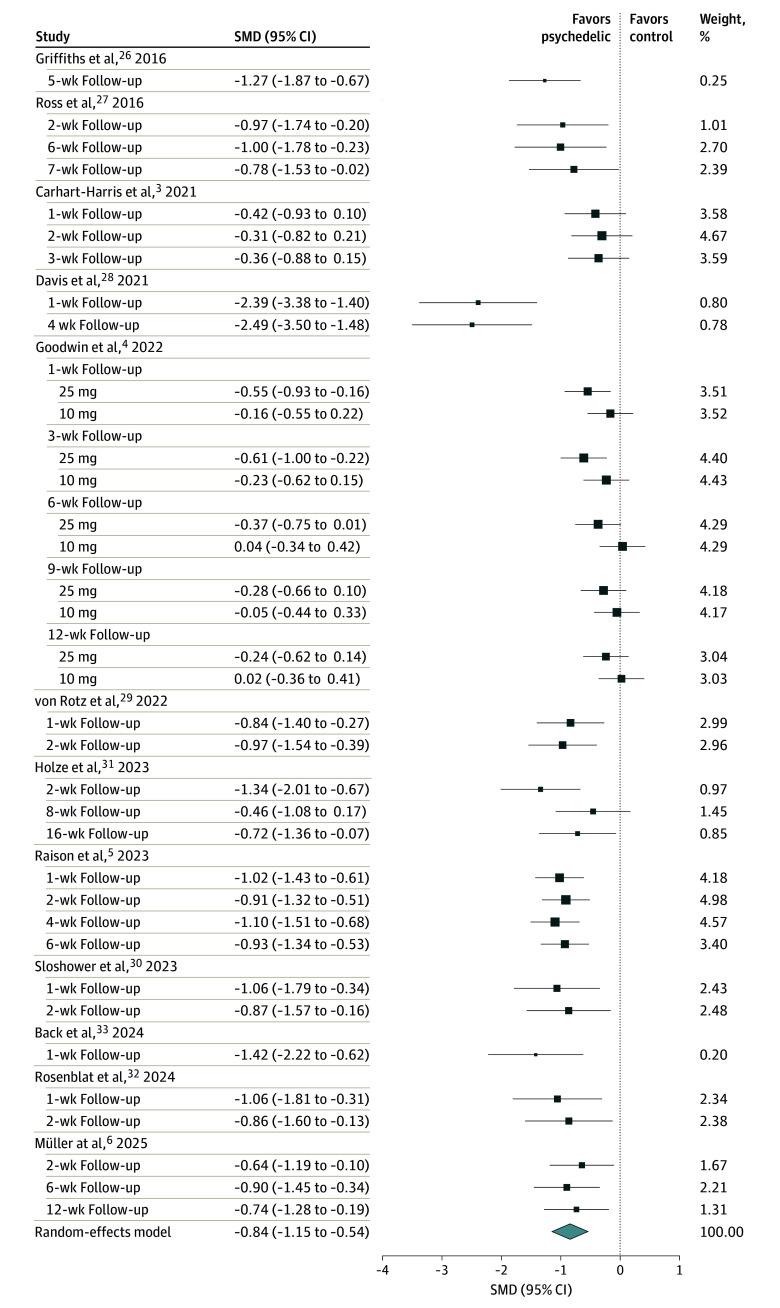
Forest Plot of Meta-Analysis Results Depicted are standardized mean differences (SMDs) and 95% CIs for all studies included in meta-analysis (36 effect sizes from 12 trials). Follow-up time point is indicated in weeks from the last dosing session. Negative values indicate larger reduction in depressive symptoms in treatment condition. The pooled effect size (denoted by the diamond) was estimated using robust variance estimation. The size of points indicates their relative weight in the model.

The multilevel meta-analysis revealed a significant and large depressive symptom reduction in PAT compared with control conditions (Hedges *g* = −0.84; 95% CI, −1.15 to −0.54; *P* < .001) (Figure 2). Residual heterogeneity was substantial (*Q_e_*_35_ = 94.47; *P* < .001), with total *I*^2^ of 72.17% (between-study = 69.08%; within-study = 3.09%).

### Metaregression Analyses: Psychological Therapy Variables

More preparation hours were associated with larger depressive symptom reduction. In the univariate metaregression with 36 effect sizes, each additional hour of preparation was associated with decreased depressive symptoms (β = −0.13; 95% CI, −0.24 to −0.01; *P* = .04). All other psychological therapy variables, namely hours of integration (β = −0.02; 95% CI, −0.08 to 0.05; *P* = .53), number of sessions (β = −0.01; 95% CI, −0.09 to 0.08; *P* = .86), and total duration in weeks (β = −0.01; 95% CI, −0.02 to 0.01; *P* = .46) were not significantly associated with treatment outcomes in univariate regression models.

### Exploratory Metaregression Analyses

Longer follow-up intervals were associated with less symptom reduction (33 effect sizes; β = 0.02; 95% CI, 0.01-0.04; *P* = .003). All other covariates, including number of dosing sessions, baseline depression severity, participant age, sex, publication year, and scale rater type were not significantly associated with symptom reduction (eTable 1 in Supplement 1).

### Sensitivity Analyses

Davis et al^28^ exhibited high Cook distance (using a threshold of 0.5) in the preparation hours regression model (*D* = 0.94), indicating a disproportionate influence. Excluding this study leaving 34 effect sizes led to a smaller pooled treatment effect size (*g* = −0.74). The significance of preparation hours and follow-up duration models did not change after exclusion.

Excluding studies with waiting list and active treatment control groups leaving 29 effect sizes reduced the overall effect size (*g* = −0.78). Preparation hours and follow-up duration regression models remained significant. We additionally varied assumed within-study autocorrelation (φ) across a range of different values, the results of which can be found in eTable 2 in Supplement 1.

### Publication Bias

**Figure 3.**
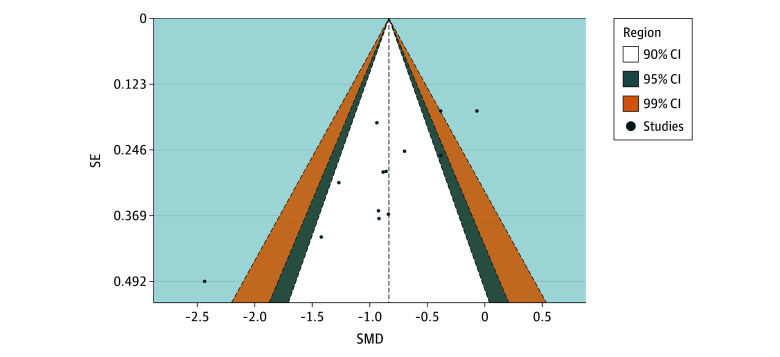
Funnel Plot Assessing Publication Bias Among Studies Vertical line indicates the pooled estimated effect size using robust variance estimation. Each dot represents the aggregated effect size per included study condition to enhance legibility. SMD indicates standardized mean difference.

Visual inspection of the funnel plot (Figure 3) showed asymmetry, with Egger test indicating small-study effects (*z* = −4.09; *P* < .001). In the funnel plot, both treatment arms of Goodwin et al^4^ and Davis et al^28^ were outside of the 95% CI. Trim-and-fill imputed 5 missing studies, with the pooled treatment effect size decreasing to *g* = −0.59 (95% CI, −0.91 to −0.28; *P* < .001). Although this indicates possible small-study bias, the antidepressant outcomes associated with PAT remained moderate to large and robust to correction.

### Risk-of-Bias Assessment

Nine studies were rated as having a high risk of bias, with the other 3 being rated as low. High bias ratings were primarily related to ineffective blinding of participants and clinicians, which is a frequent problem in interventions using psychedelic compounds. A summary of risk of bias assessments is presented in eFigures 1 and 2 in Supplement 1.

## Discussion

The goal of this systematic review and meta-analysis was to assess whether the quantity of psychological therapy is associated with outcomes in PAT for depressive symptoms. In total, 12 controlled trials (733 participants) were pooled for meta-analysis. Overall, PAT showed significantly greater depressive symptoms reduction compared with control conditions, which is in line with estimates from previous meta-analyses.^34,35,36,37,38^ Regression analyses revealed a significant association between more hours of preparation and larger symptom reductions, but no association with integration hours or total treatment duration. Longer follow-up periods were significantly associated with smaller treatment effect sizes. Important to note is that these results pertain to quantitative aspects of therapy exposure rather than qualitative or process-related dimensions of the therapeutic interaction, which likely contribute substantially to outcomes. Such qualitative aspects may include the strength of the therapeutic alliance, empathic attunement, and therapist presence, as well as broader psychotherapeutic change mechanisms like resource activation, therapeutic relationship, problem actuation, clarification, and mastery.^39,40,41,42,43^ In line with this, recent meta-analytic evidence suggests that therapeutic alliance in PAT may be as important as the drug itself.^40^

The suggested association between preparation amount and treatment outcomes could reflect the role of psychological framing prior to dosing. The goal in preparation sessions is to establish a strong therapeutic alliance, clarify intentions, reduce anxiety, and to create trust in the treatment process. They also provide time to cultivate favorable motives for the dosing session, such as an approach-oriented focus on exploring and working on personal challenges. This has been shown to be associated with better treatment outcomes (compared with avoidance-orientation) in both psychedelic use and conventional psychotherapy.^44,45^ Spending more time on preparation could, therefore, not only reduce uncertainty but also increase psychological readiness, openness, and emotional engagement during dosing sessions, thus maximizing their therapeutic benefit. This aligns with the longstanding theory in the psychedelic field stressing the importance of “set and setting.”^46^ It is also consistent with Grawe’s theory that the human need for control and orientation is central to psychological stability.^47^ Within this framework, thorough preparation can enable a more stable internal orientation when faced with the intense and often unpredictable nature of psychedelic states, thereby increasing the likelihood of a constructive, emotionally integrative process, and positive treatment outcomes. Additionally, empirical findings have consistently shown that certain qualitative features of the psychedelic experience (eg, subjective intensity) are associated with better therapeutic outcomes.^48,49,50^ Along this line of reasoning, extensive preparation could help facilitate a more therapeutically valuable psychedelic experience, which then, in turn, leads to a larger reduction of depressive symptoms.

These findings differ from those of a recent meta-analysis,^51^ which found no significant associations for psychological therapy variables, likely due to methodological differences. Their analysis also included open-label psilocybin trials, which can produce inflated effect sizes due to selection bias, small sample sizes, and lack of control conditions. Pooling with controlled studies may obscure associations between therapy quantity and outcome. In contrast, we focused exclusively on standardized mean differences from controlled trials, which provides a more conservative estimate of treatment outcomes.

Somewhat surprisingly, other psychological therapy variables, such as integration hours or total treatment duration, were not significantly associated with better treatment outcomes. This could imply that not all therapeutic input contributes equally or that outcomes are nonlinear or phase specific. While preparation may influence acute outcomes the most, integration could help with consolidation and thus more sustained symptom reduction. Therefore, the nonsignificant findings for integration should be interpreted cautiously, as the lack of significant association could be caused by methodological constraints. Integration impacts could only emerge after longer time frames, or on broader outcomes such as functioning, quality of life, or meaningfulness, which are rarely captured. Benefits may also be more dependent on specific therapeutic practices or skills compared with preparation. It is also plausible that the amount of integration provided in these trials (mean 4.4 hours) was not sufficient to have a measurable impact.

Unsurprisingly, longer follow-up durations were linked to smaller symptom reductions, likely reflecting natural decay in psychedelic treatment outcomes. As mentioned previously, sustaining the treatment benefit could require ongoing integration sessions in addition to periodic booster dosing sessions. We believe that future trials should aim to standardize follow-up intervals and examine symptom trajectories over longer time frames to better understand maintenance and relapse patterns.

Beyond the psychedelic field, our findings can also be situated within the broader landscape of psychotherapy research, where greater session quantity is generally linked to larger symptom improvements, with most change occurring in earlier sessions.^52^ Additionally, common factors like the therapeutic alliance has consistently been shown as cross-modal factor associated with outcome in all treatment phases.^53^ Framing PAT within this broader psychotherapy literature could suggest that therapy quantity in PAT may partly influence these common mechanisms of change, highlighting the necessity for closer integration between psychedelic and psychotherapy research.

The broader implication of these findings is that the efficacy of psychedelic treatment models is probably shaped substantially by the therapeutic context in which it is embedded. Compared with most conventional pharmacological treatments, psychedelics appear to exert their effects through a complex interplay of both psychological and biological factors. This is both an opportunity and a challenge. On one hand, it can offer patients a more individualized model of care. On the other hand, it complicates efforts to standardize and scale these interventions for broader clinical implementation. Regulatory and training frameworks for clinical application will remain difficult to define if we cannot develop evidence-based guidelines on essential or optimal psychological therapy components in PAT. Still, regulatory frameworks must also be able to balance a certain flexibility for therapist judgment with evidence-based minimum standards.

To advance the field, future research should employ more rigorous therapy-focused designs and focus on more transparent reporting. Dismantling trials or factorial designs could test both the necessity and optimal quantity of specific therapy components, such as preparation, integration, or therapeutic approach. Key therapeutic elements in PAT could be identified by trials comparing high-support vs minimal-support models or manualized disorder-specific vs nondirective approaches. Ideally, studies would also include frequent long-term monitoring, standardized intervention reporting, and assessment of general psychotherapeutic change mechanisms across all phases of treatment.^41,57^ Such systematic investigations will be essential to clarify mechanisms of action and ensure that future patients benefit from evidence-based treatment options.

### Limitations

There are several limitations that should be noted. Most of the trials were rated as having a high risk of bias, mostly due to the difficulty of blinding in psychedelic trials. However, this issue is not unique to psychedelics, as similar placebo and blinding challenges occur in studies of conventional antidepressants or psychotherapy.^54^ The absence of formal preregistration is an additional limitation, as it may increase the risk of reporting bias; however, all inclusion criteria, data extraction procedures, and analyses were predefined in an internal protocol (eMethods in Supplement 1), and we do not anticipate that this materially affected the results. Another limitation is the inadequate and inconsistent reporting, as well as the unsystematic application of psychological therapy components in current PAT trials.^55^ Most of the included studies did not measure or report other highly relevant aspects of the psychological intervention (eg, session content, theoretical orientation, or therapy manual). Therefore, determining whether certain therapeutic approaches or protocol features are more impactful remains an open question. Additionally, many trials lacked a systematic therapeutic approach, often involving therapists from diverse professional and theoretical backgrounds without a clear treatment manual. Some trials also allowed variable durations of therapy sessions, which may have introduced additional variability in treatment exposure and contributed to heterogeneity in our primary analyses. The generalizability of these findings is constrained by the small number of studies currently available, homogeneity of participant demographics, and use of study-level covariates, which limit individual-level inferences.^56^ Thus, results should be considered preliminary.

## Conclusions

In this systematic review and meta-analysis of controlled clinical trials investigating PAT for depressive symptoms, a greater quantity of preparation therapy was associated with significantly larger reductions of depressive symptoms. These findings suggest an important role of preparation sessions in PAT. Additionally, we confirmed previous findings demonstrating a general antidepressant potential of PAT. However, the broader role and impact of psychological therapy and its application in PAT remains insufficiently understood from a scientific point of view. Uncertainty persists not only regarding qualitative and process-related aspects of the psychological therapy but also the optimal structure and quantity of therapeutic components. Generalizations of these findings are limited by high heterogeneity, study quality, and underreporting. To advance this field we need rigorous, mechanistically informed research to clarify the role of psychological therapy. Only then can we develop and scale truly evidence-based models of PAT.

## Notes

Supplement 1. eMethods. eTable 1. Supplementary meta-regression results eTable 2. Sensitivity analysis of assumed within-study correlation eFigure 1. Risk of bias in randomized trials (RoB-2) eFigure 2. Risk of bias in nonrandomized trials (ROBINS-I) eReferences.

Supplement 2. Data Sharing Statement
