# Supplementary material for: Psychological Therapy Quantity and Depressive Symptom Reduction in Psychedelic-Assisted Therapy: A Systematic Review and Meta-Analysis
Source: JAMA Netw Open. 2026 Jan 21;9(1):e2554843. doi: 10.1001/jamanetworkopen.2025.54843 (PMC12824788; doi:10.1001/jamanetworkopen.2025.54843)
Supplement: Supplement 1. — eMethods. eTable 1. Supplementary meta-regression results eTable 2. Sensitivity analysis of assumed within-study correlation eFigure 1. Risk of bias in randomized trials (RoB-2) eFigure 2. Risk of bias in nonrandomized trials (ROBINS-I) eReferences. [file jamanetwopen-e2554843-s001.pdf]

Supplemental Online Content

Florineth GA, Klima I, Boeker AL, et al. Psychological therapy quantity and depressive symptom reduction in psychedelic-assisted therapy. *JAMA Netw Open.* 2026;9(1):e2554843. doi:10.1001/jamanetworkopen.2025.54843

eMethods.

eTable 1. Supplementary meta-regression results

eTable 2. Sensitivity analysis of assumed within-study correlation

eFigure 1. Risk of bias in randomized trials (RoB-2)

eFigure 2. Risk of bias in nonrandomized trials (ROBINS-I)

eReferences

This supplemental material has been provided by the authors to give readers additional information about their work.

© 2026 Florineth GA et al. *JAMA Network Open*.

eMethods

Data extraction

If standard errors were reported instead of standard deviations, we converted them according to Cochrane guidelines. Where data were not available in the manuscript or supplement, authors were contacted via e-mail for clarification. If unsuccessful, data available only in figures were extracted graphically using the well-validated WebPlotDigitizer software[1]. Any data still missing were not analyzed.

Search string (PubMed)

((trial[Title]) OR (placebo controlled[Title]) OR (randomized controlled [Title]) OR (double-blind[Title/Abstract]))

AND ((psilocybin[Title/Abstract]) OR (lysergic acid diethylamide[Title/Abstract]) OR (mescaline[Title/Abstract]) OR (dimethyltryptamine [Title/Abstract]) OR (ayahuasca[Title/Abstract]))

AND ((depression[Title/Abstract]) OR (life-threatening disease[Title/Abstract]))

NOT ((animal[Title/Abstract]) OR (healthy[Title]) OR (review[Title]) OR (meta-analysis[Title]) OR (microdosing[Title]) OR (proof-of-concept[Title]) OR (protocol[Title]) OR (corrigendum[Title]) OR (open label[Title])OR (case report[Title]) OR (mdma[Title]) OR (review[Publication Type]))

This search string was adapted in other databases to fit specific requirements retaining the same search terms.

Preregistration

A study protocol was not preregistered. However, an internal study protocol was created before the initiation of the review process and adhered to throughout the entire process. We have uploaded this protocol on OSF: https://doi.org/10.17605/OSF.IO/3NC7Z .

Excluded studies of note

"Greater subjective effects of a low dose of LSD in participants with depressed mood" by Molla et al. (2024)[2] was excluded as only a very low dose of LSD (26 micrograms) was used, which is not expected to elicit a full spectrum of psychedelic effects.

"Pilot Study of Psilocybin Treatment for Anxiety in Patients With Advanced-Stage Cancer" by Grob et al. (2011)[3] was excluded as any information regarding the quantity of psychological therapy provided could not be obtained.

© 2026 Florineth GA et al. *JAMA Network Open*.

eTable 1. Supplementary meta-regression results

Variable	*β*	95% CI	p-value
**number of dosing sessions**	-0.123	-0.824 to 0.577	0.706
**mean age**	-0.014	-0.05 to 0.021	0.394
**sex**	0.001	-0.031 to 0.034	0.923
**depression scale category**	0.302	-0.26 to 0.863	0.262
**baseline depression severity**	-0.188	-0.678 to 0.303	0.414

eTable 2. Sensitivity analysis of assumed within-study correlation

		Φ -1	Φ -0.75	Φ -0.5	Φ -0.25	Φ 0	Φ 0.25	Φ 0.5	Φ 0.75	Φ 1
**pooled effect size**	*g*	-0.916	-0.890	-0.879	-0.877	-0.879	-0.879	-0.875	-0.861	-0.836
**meta-regression: hours preparation**	*β*	-0.161	-0.141	-0.135	-0.134	-0.136	-0.137	-0.137	-0.132	-0.133
	p	0.046	0.055	0.050	0.046	0.044	0.042	0.040	0.039	0.023
**meta-regression: weeks follow-up**	*β*	0.024	0.024	0.026	0.027	0.027	0.027	0.027	0.026	0.026
	p	0.022	0.002	0.001	0.001	0.001	0.001	0.001	0.001	0.004

© 2026 Florineth GA et al. *JAMA Network Open*.

eFigure 1. Risk of bias in randomized trials (RoB-2)

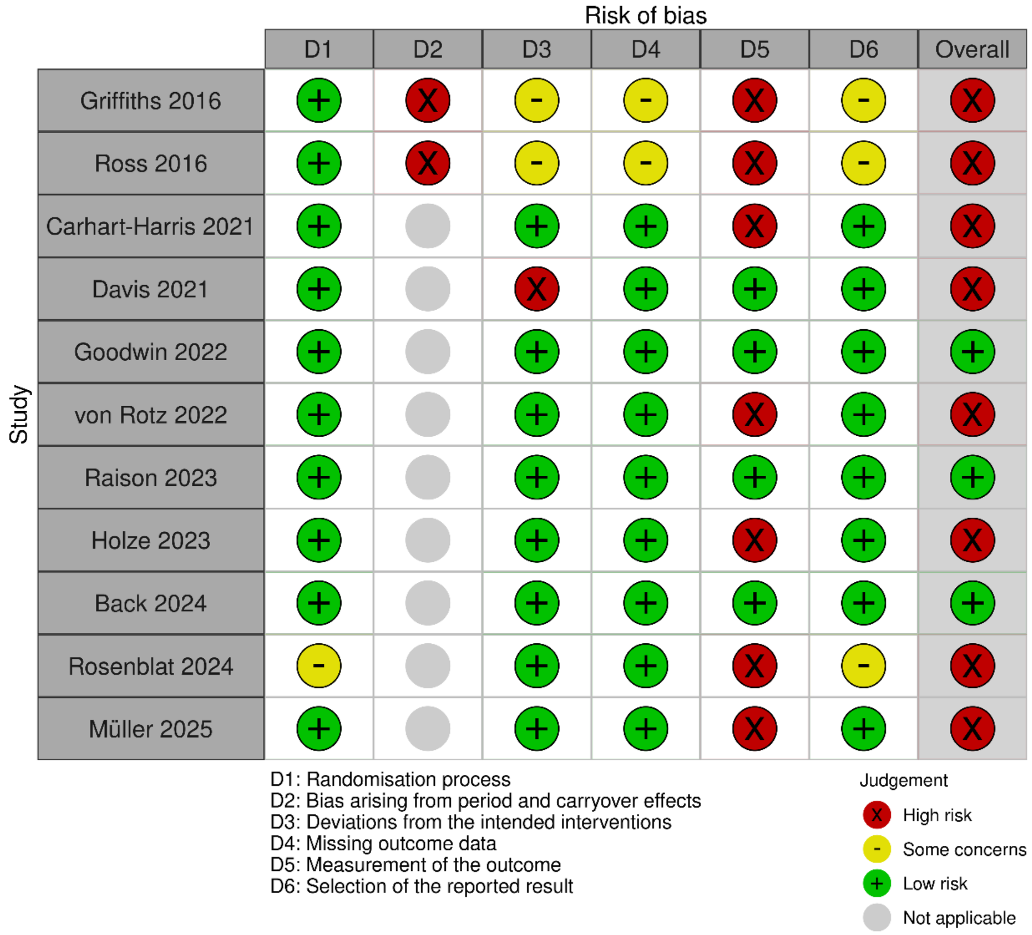

eFigure 2. Risk of bias in nonrandomized trials (ROBINS-I)

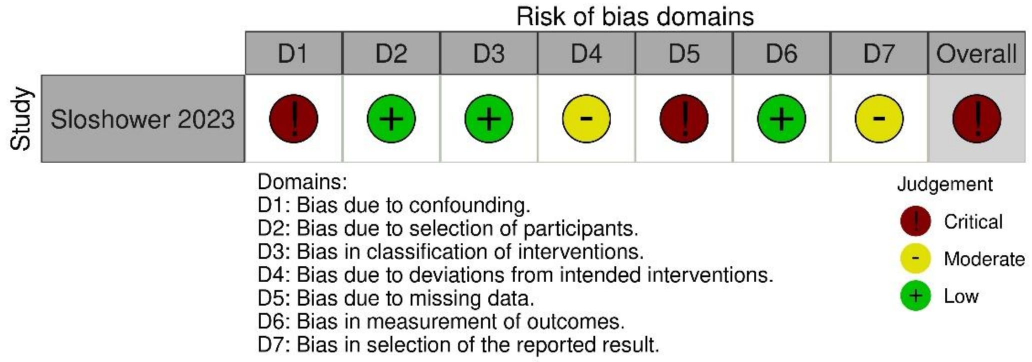

eReferences

1. Rohatgi A. WebPlotDigitizer. Published online 2024. Accessed June 30, 2025. https://automeris.io

2. Molla H, Lee R, Tare I, de Wit H. Greater subjective effects of a low dose of LSD in participants with depressed mood. *Neuropsychopharmacol Off Publ Am Coll Neuropsychopharmacol*. 2024;49(5):774-781. doi:10.1038/s41386-023-01772-4

3. Grob CS, Danforth AL, Chopra GS, et al. Pilot study of psilocybin treatment for anxiety in patients with advanced-stage cancer. *Arch Gen Psychiatry*. 2011;68(1):71-78. doi:10.1001/archgenpsychiatry.2010.116

© 2026 Florineth GA et al. *JAMA Network Open*.
