# Supplementary material for: Psychological Therapy Quantity and Depressive Symptom Reduction in Psychedelic-Assisted Therapy: A Systematic Review and Meta-Analysis
Source: JAMA Netw Open. 2026 Jan 21;9(1):e2554843. doi: 10.1001/jamanetworkopen.2025.54843 (PMC12824788; doi:10.1001/jamanetworkopen.2025.54843)
Supplement: Supplement 2. — Data Sharing Statement [file jamanetwopen-e2554843-s002.pdf]

Data Sharing Statement

Florineth. Psychological Therapy Quantity and Depressive Symptom Reduction in Psychedelic-Assisted Therapy. *JAMA Netw Open*. Published January 21, 2026.
doi:10.1001/jamanetworkopen.2025.54843

Data
Data available: Yes
Data types: Other (please specify)
Additional Information: Study-level data extracted from the original publications included in the meta-analysis
How to access data: gianluca.florineth@unibe.ch
When available: With publication

Supporting Documents
Document types: Statistical/analytic code, Other (please specify)
Additional Information: Study Protocol
How to access documents: Study protocol: https://doi.org/10.17605/OSF.IO/3NC7Z
Statistical code: request from gianluca.florineth@unibe.ch
When available: With publication

Additional Information
Who can access the data: researchers whose proposed use of the data has been approved
Types of analyses: for any purpose
Mechanisms of data availability: data will be made available after approval of a proposal
